# Association of protein tyrosine phosphatase non-receptor type 22 gene functional variant C1858T, HLA-DQ/DR genotypes and autoantibodies with susceptibility to type-1 diabetes mellitus in Kuwaiti Arabs

**DOI:** 10.1371/journal.pone.0198652

**Published:** 2018-06-20

**Authors:** Mohammad Z. Haider, Majedah A. Rasoul, Maria Al-Mahdi, Hessa Al-Kandari, Gursev S. Dhaunsi

**Affiliations:** 1 Department of Pediatrics, Faculty of Medicine, Kuwait University, Jabriya, Kuwait; 2 Department of Pediatrics, Adan Hospital, Al-Adan, Kuwait; 3 Department of Pediatrics, Farwania Hospital, Farwania, Kuwait; 4 Medical Laboratories, Mubarak Al-Kabeer Hospital, Jabriya, Kuwait; Charles University, CZECH REPUBLIC

## Abstract

The incidence of type-1 Diabetes Mellitus (T1DM) has increased steadily in Kuwait during recent years and it is now considered amongst the high-incidence countries. An interaction between susceptibility genes, immune system mediators and environmental factors predispose susceptible individuals to T1DM. We have determined the prevalence of protein tyrosine phosphatase non-receptor type 22 (*PTPN22*) gene functional variant (C1858T; R620W, rs2476601), HLA-DQ and DR alleles and three autoantibodies in Kuwaiti children with T1DM to evaluate their impact on genetic predisposition of the disease. This study included 253 Kuwaiti children with T1DM and 214 ethnically matched controls. The genotypes of *PTPN22* gene functional variant C1858T (R620W; rs2476601) were detected by PCR-RFLP method and confirmed by DNA sequencing. HLA-DQ and DR alleles were determined by sequence-specific PCR. Three autoantibodies were detected in the T1DM patients using radio-immunoassays. A significant association was detected between the variant genotype of the *PTPN22* gene (C1858T, rs2476601) and T1DM in Kuwaiti Arabs. HLA-DQ2 and DQ8 alleles showed a strong association with T1DM. In T1DM patients which carried the variant TT-genotype of the *PTPN22* gene, 93% had at least one DQ2 allele and 60% carried either a DQ2 or a DQ8 allele. Amongst the DR alleles, the DR3-DRB5, DR3-3, DR3-4 and DR4-4 showed a strong association with T1DM. Majority of T1DM patients who carried homozygous variant (TT) genotype of the *PTPN22* gene had either DR3-DRB5 or DRB3-DRB4 genotypes. In T1DM patients who co-inherited the high risk HLA DQ, DR alleles with the variant genotype of *PTPN22* gene, the majority were positive for three autoantibodies. Our data demonstrate that the variant T-allele of the *PTPN22* gene along with HLA-DQ2 and DQ8 alleles constitute significant determinants of genetic predisposition of T1DM in Kuwaiti children.

## Introduction

Type 1 diabetes mellitus (T1DM) has a multifactorial etiology and is considered to result from T cell-mediated beta-cell destruction in pancreas of the genetically susceptible individuals. The incidence rate of T1DM has increased rapidly in the Arabian Gulf region and globally is predicted to double in children under 5 years of age by 2020 [1]. It has been suggested that T1DM is caused by an autoimmune process involving both genetic and environmental factors and results in the destruction of insulin-producing pancreatic beta cells [2]. The genetic component to T1DM onset has been studied extensively during the last few decades [3]. These studies showed human leukocyte antigen (HLA) region as the main locus associated with T1DM susceptibility [3–4]. By using various approaches, >60 loci have been identified which are involved in genetic susceptibility to T1DM [2, 5–12]. In many of these ‘susceptibility loci’, the underlying causative genes, and/or the molecular mechanism by which they confer susceptibility are not yet known. In spite of rapid progress made by the genetic association studies, much remain to be elucidated about contribution of the susceptibility loci in different populations/ethnic groups.

Amongst the non-HLA candidate genes for T1DM, the protein tyrosine phosphatase non-receptor type 22 (*PTPN22*) gene has received much attention [13]. It is located on chromosome 1p13.3-p13.1 and encodes a lymphoid specific phosphatase (LYP) which is thought to be involved in negative control of T-cell activation and development [14]. The C1858T polymorphism in the *PTPN22* gene (rs2476601) causes an amino acid substitution at codon 620 from Arginine (Arg) to tryptophan (Trp). Previous data has shown that this polymorphism is located in the Proline-rich region of LYP and affects its catalytic activity [15]. It has been reported that the LYP-R620W variant causes a gain of physiological function and the 620W variant has more negative regulatory activity than the R620 molecule [16–17]. Bottini et al. described an association between *PTPN22* gene polymorphism (C1858T, rs2476601) and susceptibility to T1DM [18] and this association has since been confirmed in some other populations [19–24]. Previous reports revealed that there was a sharp decrease in the frequency of the variant 620W allele from Northern to Southern Europe, i.e. from around 12.5% in the English and Finnish populations, to approximately 6% in the Italian and Spanish populations [18, 25–26]. The variant 1858T allele was reported to be absent from Asian populations [27–28]. Kawasaki et al. described heterozygosity of another polymorphism (rs2488457) in the promoter region of *PTPN22* gene (-1123G/C) to be associated with acute-onset T1DM in Japanese population [29]. In Caucasians, this polymorphism is strongly linked to the R620W (rs2476601), and several studies have revealed R620W (rs2476601) to be the actual susceptibility variant [30–31]. A more recent report has compiled the association of four *PTPN22* gene polymorphisms with a number of diseases of autoimmune etiology from 21 countries and has shown that the odds ratios for -1123G/C polymorphism were considerably lower than those of rs2476601 in many populations [32]. In another recent study from Brazil, a review of the allele frequency of *PTPN22* rs2476601 polymorphism from different countries has been presented which lends support to its role as a T1DM susceptibility locus [33].

In spite of a large number of reports in the literature which show that HLA class II genes play the most important role in T1DM susceptibility, variation at these loci alone cannot explain all of the evidence of genetic association and linkage of MHC region with T1DM [34]. It has been shown that T1DM is strongly associated with HLA-DR3DQ2 and HLA-DR4-DQ8 haplotypes, alone or in combinations [35]. Nearly 90% of children diagnosed with T1DM in Scandinavia had one or both of these haplotypes [36–38]. We have previously reported association of HLA-DQB1, DQA1 and DRB1 alleles with T1DM in Kuwaiti Arab children and have identified the high risk alleles [39]. It has been shown that children homozygous for the high risk HLA-DR3-DQ2 haplotype often developed glutamic acid decarboxylase autoantibodies (GADA) first [40–41], whereas in children with the HLA-DR4-DQ8 haplotype, insulin (INS) autoantibodies were the first to appear [40–42]. Recent Next-generation- sequencing (NGS) studies revealed that HLA-DRB3, HLA-DRB4 and HLA-DRB5 are associated with beta-cell autoantibodies and therefore contribute to the increased risk of developing T1DM [43–44]. The studies which explore impact of multiple genetic risk factors e.g. *PTPN22* gene variant (C1858T, rs2476601), HLA alleles and autoantibodies in conjunction are relatively few and to our knowledge, so far there has been none from Gulf Arabs. We report the prevalence of *PTPN22* gene C1858T variant (rs2476601), HLA-DQ and DR alleles and their correlation with the presence of three autoantibodies in Kuwaiti Arabs with T1DM and controls and have evaluated their possible contributions in determining the genetic predisposition to type 1 diabetes.

## Patients and methods

This study included 253 newly diagnosed T1DM patients which were recruited from three major hospitals (Mubarak Al-Kabeer, Adan and Farwania). A sub-set of T1DM patients from this report had been included in our earlier study on the serum Vitamin D status in T1DM patients [45]. The details of patient diagnosis and recruitment have been described previously and was based on the criteria recommended in ISPAD protocol [46–47]. The information on gender, age and other characteristics of the study subjects has been presented in Table 1. The T1DM patient group was divided into three sub-groups on the basis of age-of-onset of the disease 0-4y, 4-6y and 6-14y as previously reported [47]. HbA1c was used to determine the glycemic status of the study subjects. In the T1DM patient group, 194/253 (77%) had their HbA1c between 7–10% and in 59/253 (23%) it was >10% (Table 1). A total of 214 non-diabetic controls were also studied in addition to the T1DM patients. The selection of control subjects was random, they were Kuwaiti Nationals and had comparable general characteristics to the T1DM patients. The controls were healthy volunteers and their health status was evaluated by a specialist. All the controls had their HbA1c below 6.5%. This study was approved by the Joint Ethics Committee of the Faculty of Medicine, Kuwait University and the Kuwait Institute of Medical Specializations, Ministry of Health, Kuwait. Written informed consent was obtained from the participants and/or their parents according to the prescribed rules of the Ethics Committee.

**Table 1:** Characteristics of Kuwaiti T1DM patients (n = 253) and controls (n = 214).

	T1DM Patients	Controls	P-value	OR*	95% CI**
**Gender**					
Males	124	110	0.50	0.84	0.575–1.241
Females	129	104	0.51	1.062	0.792–1.691
**Age** **(yrs., mean ± SD)**					
	8.5 (±5.5)	8.9 (±5.2)	NS		
**HbA1c**					
7–10%	194/253 (77%)	0			
>10%	59/253 (23%)	0			
<6.5%	0	100%			
Ethnicity*					
Kuwaiti	100%	100%	NS		
**Age onset of T1DM (No of subjects, %)**					
< 4 yrs.	51 (20%)				
4–6 yrs.	71 (28%)				
>6 yrs.	131 (52%)				

*OR, Odds ratio;

**95% CI, 95% confidence interval; yrs., years, mean+SD, mean, standard deviation; N.S., not significant;

^a^All the study subjects (T1DM patients and controls) were Kuwaiti Nationals resident in three large population density areas of the country at the time of the study.

### Sample collection and processing

The details of sample collection and processing have been described previously [45]. Briefly, “approximately 5 ml blood was collected from T1DM patients and controls in appropriate tubes. The blood was drawn from patients upon first diagnosed of T1DM, the serum was isolated from samples collected in plain tubes and stored at -30°C for subsequent analyses [45]”. For genotyping studies, blood was anti-coagulated with EDTA. The total genomic DNA was extracted by a previously described method [48]. The concentration of Hemoglobin A1c (HbA1c) was determined by high performance liquid chromatography (HPLC).

### Identification of genotypes for *PTPN22* receptor gene functional variant (C1858T)

**Fig 1:**
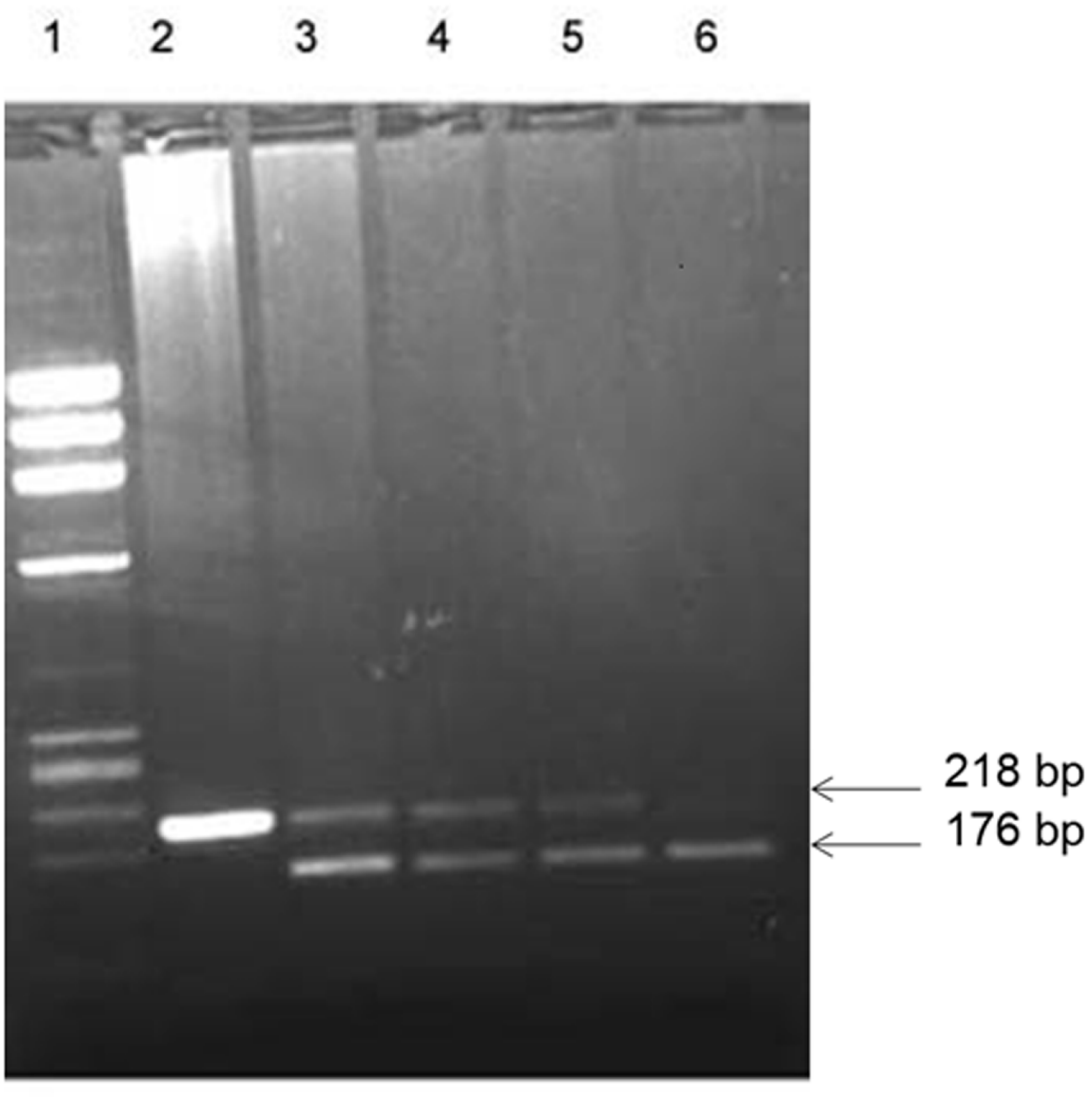
Detection of *PTPN22* gene functional variant [C1858T; rs2476601] genotypes. PCR amplification of genomic DNA was carried out (details given in Patients and methods) and products of amplification were cleaved with restriction enzyme *Rsa*I. Lane 1, phiX174 *Hae*III cut *M*_*r*_ markers; lane 2, products from subjects having TT genotype; lanes 3–5, products from subjects having CT genotype; lane 6, products from subject with CC genotype. The numbers on the side are sizes of the characteristic bands resulting from PCR-RFLP analysis of subjects with different genotypes. The restriction enzyme cleavage products were analyzed on 2% agarose gel and visualized under UV light after staining with ethidium bromide.

The genotypes for a non-synonymous single nucleotide polymorphism (SNP) +1858C→T (rs2476601) in the *PTPN22* gene were identified by polymerase chain reaction-restriction enzyme fragment length polymorphism (PCR-RFLP) method as described earlier [49]. A 218 bp DNA fragment was amplified by using the primers: Forward primer: 5’- ACTGATAATGTTGCTTCAACGG-3’ and reverse primer: 5’-TCACCAGCTTCCTCAACCAC-3’. The PCR mixture consisted of 10x PCR buffer (Applied BioSystems, Foster City, USA); 1.5 mM MgCl_2_; 0.2 mM of each of the dNTPs (deoxyribonucleotide triphosphates); 20 pmol of each primer, 250 ng template DNA and 1U AmpliTaq DNA polymerase (Applied BioSystems). The amplification conditions used were denaturation at 94°C for 2 min followed by 35 cycles of 94°C for 30 seconds, 60°C for 30 seconds and 72°C for 30 seconds and an extension step at 72°C for 5. The PCR product was digested with restriction enzyme *Rsa*I at 37°C for 90 min. The cleavage products were analyzed by 2% agarose gel electrophoresis and visualized under UV light after staining with ethidium bromide. The *Rsa*I restriction enzyme site was absent from the 218 bp PCR product in the case of 1858T allele, while in the 1858C allele was associated with the presence of 176 bp and 42 bp cleavage products. In a heterozygous individual, both the 218 and 176 bp bands were present. An example of genotype-detection is presented in Fig 1. The PCR amplicons were sequenced on ABI 3130 genetic analyzer to confirm the genotypes.

### Detection of HLA DQ and DR alleles

The analysis of HLA-DQ and DR alleles was carried out in selected Kuwaiti T1DM patients. The selection was made on the basis of the subjects carrying one of the three genotypes of the *PTPN22* gene (i.e. CC, CT and TT). Appropriate number of subjects were chosen with these three genotypes for analysis of HLA-DQ and DR alleles. For determination of HLA-DQ and DR alleles, PCR-SSP (polymerase chain reaction-sequence specific primers) method was used [50]. DYNAL DQ and DR “Low resolution”-SSP kits (Dynal, Oslo, Norway) were used to detect the HLA-DQ and DR alleles in T1DM patients. The PCR conditions and subsequent analysis was carried out as described in the kit’s instructions manual. The alleles were determined by using the “Interpretation Table” supplied with the kits.

### Detection of autoantibodies

Three autoantibodies, Islet Cell autoantibody (ICA), glutamic acid decarboxylase (GADA) and Insulin (INS) were detected in the serum samples from T1DM patients by radioimmunoassay using commercial kits (Cisbio Assays, Codolet, France; www.cisbio.com). Protein-A Sepharose coated 96-well filtration plate was used to collect the antigen-antibody immune-complexes. The ^35^S beta emission was estimated on a liquid scintillation radioactivity counter. The cut off values used for GADA and ICA were 180 and 80 cpm/50 microlitre of sera respectively, and the patients were grouped into autoantibody positive and negative categories.

### Statistical analysis

The data was analyzed using the Statistical Package for the Social Sciences version 23 (SPSS, Chicago IL, USA). The frequencies of various genotypes and alleles detected among T1DM patients and controls were calculated by direct counting. The confidence interval (CI) was set at 95% and statistical significance was set at P <0.05 (two-tailed). Fisher’s Exact test was used to determine statistical significance of the differences between genotype and allele frequency in the T1DM patients and controls. For calculation of the statistical significance in co-dominant and dominant models, the genotype frequency in homozygous CC subjects and the ‘C’ allele frequency were considered as reference (assumed to be associated with the least risk of T1DM). In the case of dominant model, the genotype frequencies of CT and TT were pooled (T1DM patients having at least one variant allele of the *PTPN22* gene rs2476601 polymorphism). A posteriori power analysis was carried out to evaluate the strength of statistical analysis. One limitation of the study was that in some comparisons e.g. age-of-onset in T1DM patients with autoantibody-positivity and the homozygous variant (TT) genotype, the presence of low numbers in study groups may thus cause type-II errors. The genotype distribution was tested for Hardy Weinberg equilibrium by goodness of fit method using MSTAT software.

## Results

### Distribution of *PTPN22* C1858T, rs2476601 genotypes in T1DM patients and controls

The frequency of variant genotype of *PTPN22* gene (C1858T) was found to be significantly different between Kuwaiti T1DM patients and controls (Table 2). When the frequencies of the C and T allele and the *PTPN22* genotypes were compared between T1DM and control groups, the differences were statistically significant (Table 2). When the genotype frequencies of the homozygous TT and heterozygous CT subjects were pooled, 37% T1DM patients carried at least one variant ‘T’ allele compared to 16% of the controls. A posteriori power analysis revealed that at 5% significance level, the final sample size of 253 cases and 214 controls having respectively the prevalence of CT genotype as 30% and 15%, provided the study power of 97% to estimate the smallest observed effect size of 2.67 that related CT allele with the diabetes’ status of the participants. In comparison, power calculation for the TT genotype (OR 9.56) provided the study power of 100% for estimation of the diabetes risk.

**Table 2:** Frequency of *PTPN*22 gene functional variant (C1858T, rs2476601) genotypes and alleles in Kuwaiti TIDM patients and controls patients and controls (for details please see Patients and methods).

Genotype/Alleles	TIDM Patients N = 253 (%)	Controls N = 214 (%)	OR (95% CI)*	P-value**
**Co-dominant**				
CC	160 (63)	180 (84)	1.00 (Reference)^a^	
CT	76 (30)	32 (15)	2.67 (1.68–4.25)	<0.001
TT	17 (7)	2 (1)	9.56 (2.18–42.0)	<0.001
**Dominant**				
CC	160 (63)	180 (84)	1.00 (Reference)^a^	
CT/TT	93 (37)	34 (16)	3.08 (1.97–4.81)	<0.001
**Alleles**	**n = 506 (%)**	**n = 428 (%)**		
C	396 (78)	392 (92)	1.00	
T	110 (22)	36 (8)	3.03 (2.03–4.52)	<0.001

*OR (95% CI), odds ratio at 95% confidence interval;

**P-values were considered significant when <0.05.

^a^ Genotype frequency in the case of homozygous CC subjects and allele frequency of ‘C’ were considered as reference (assuming to be associated with least risk of T1DM) for calculation of statistical significance using Fisher’s Exact test.

### Detection of HLA-DQ/DR alleles in T1DM patients and their correlation with *PTPN22* genotypes

**Fig 2:**
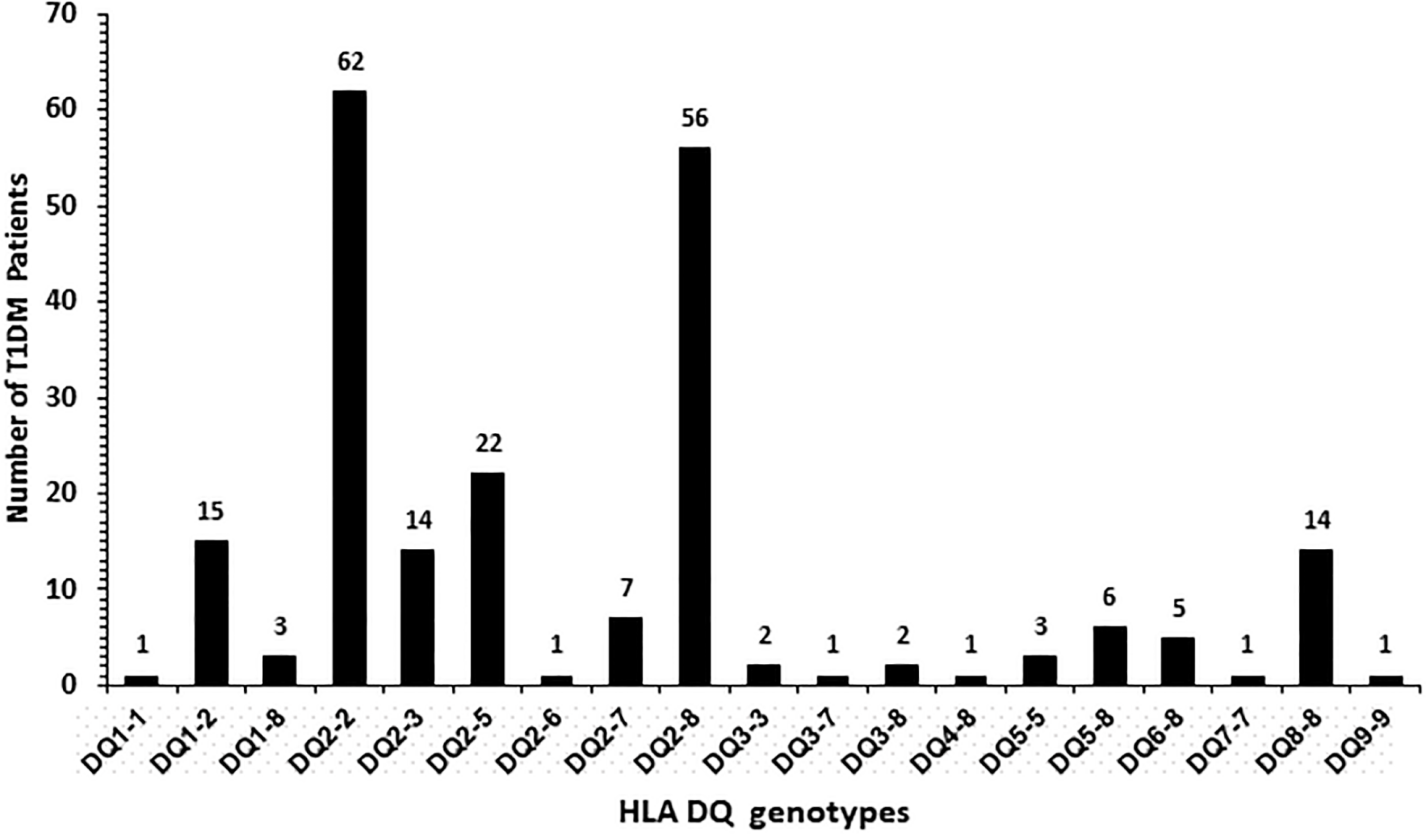
Frequency of HLA-DQ genotypes in Kuwaiti T1DM patients.

**Fig 3:**
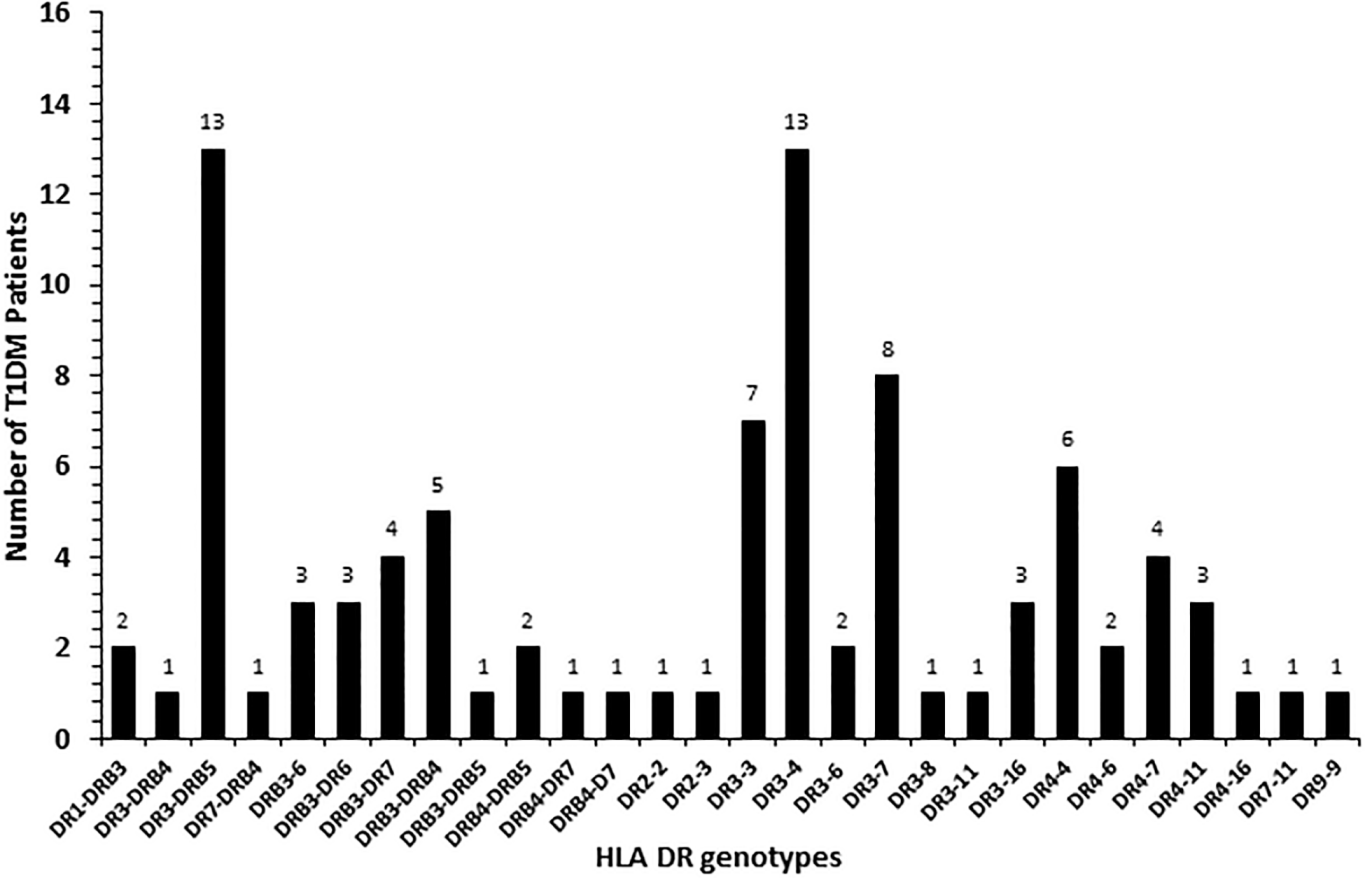
Frequency of HLA-DR genotypes in Kuwaiti T1DM patients.

**Fig 4:**
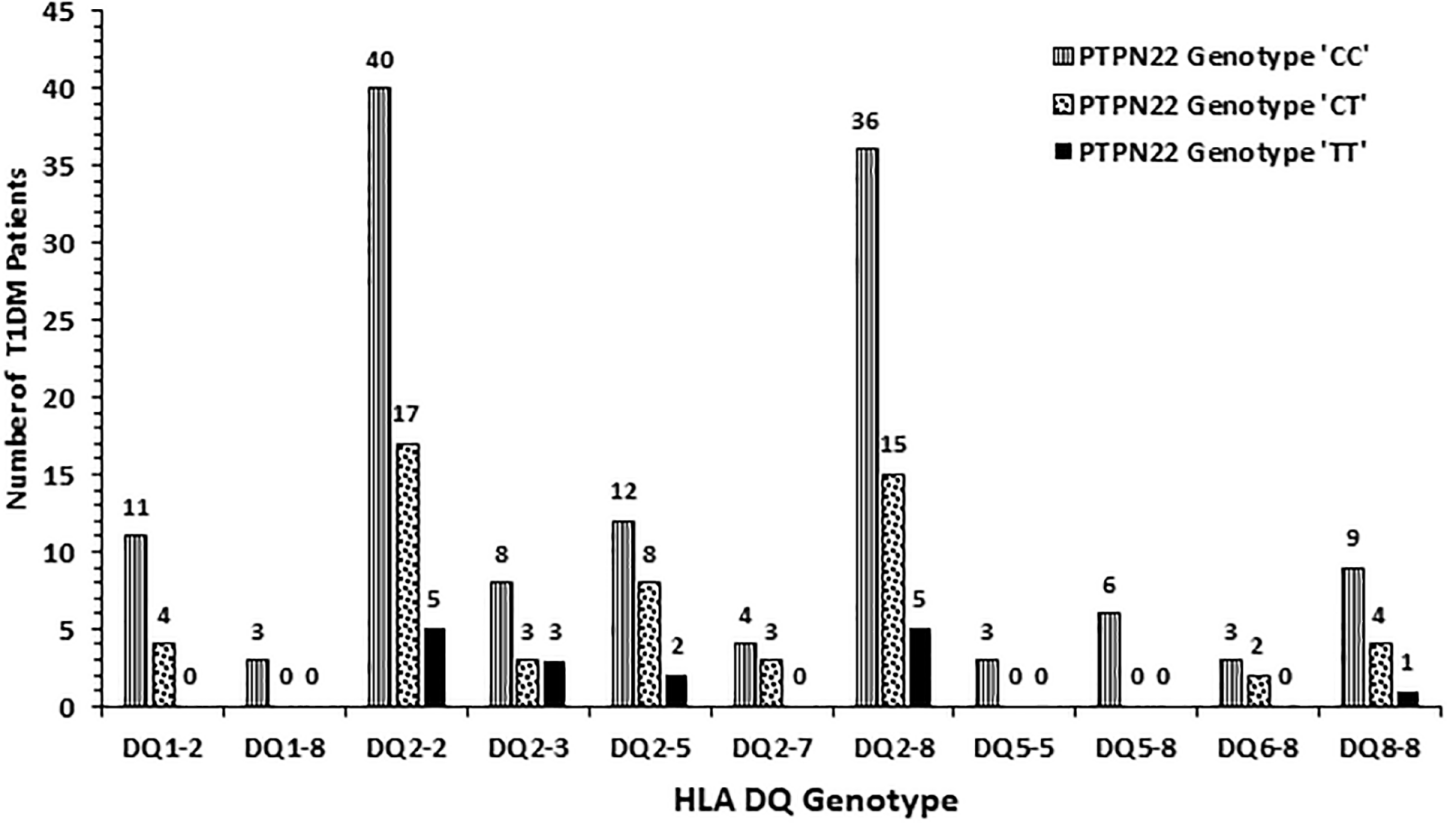
Association of HLA-DQ genotypes with genotypes of *PTPN22* gene (C1858T, rs2476601) functional variant in Kuwaiti T1DM patients.

**Fig 5:**
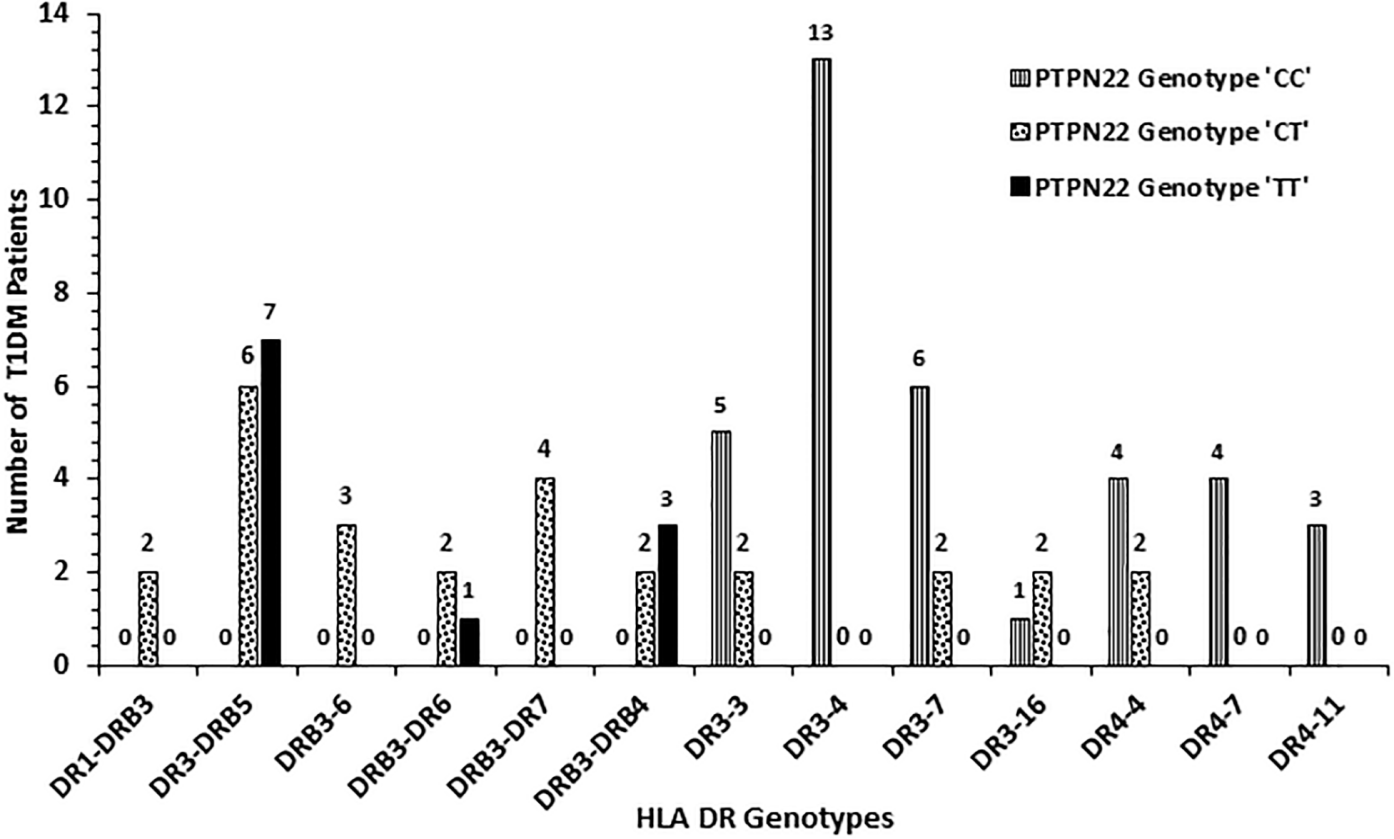
Association of HLA-DR genotypes with genotypes of *PTPN22* gene (C1858T, rs2476601) functional variant in Kuwaiti T1DM patients.

The frequency of HLA-DQ alleles in Kuwaiti T1DM patients is presented in Fig 2. Altogether nineteen different allelic combinations were detected. Six of these (DQ 1–2; 2–2, 2–3, 2–5, 2–8 and 8–8) showed a relatively high incidence. Amongst these, DQ 2–2 and DQ 2–8 had the highest incidence (Fig 2). In 98 (55%) T1DM patients, the genotype was either homozygous for DQ2 or in combination with a DQ8 allele (Fig 2). Similarly, in 58 (36%) patients, the genotype was homozygous for DQ8 or with other alleles. Collectively, 91% of all the T1DM patients studied had either DQ2 or DQ8 alleles in different combinations with other alleles (Fig 2). The incidence of HLA-DR alleles in T1DM patients has been presented in Fig 3 which showed that twenty-eight different allelic combinations were detected; of which, eight (with incidence >4) were more pronounced. HLA-DR 3–5, 3–3, 3–4, 3–7 and 4–4 showed highest incidence amongst the T1DM patients studied (Fig 3). The data on co-inheritance of HLA-DQ and the *PTPN22* genotypes in T1DM patients is presented in Fig 4. The variant, TT genotype of *PTPN22* gene was detected together with five different allelic combinations of HLA-DQ (i.e. DQ2-2, 2–3, 2–5 2–8 and 8–8 respectively, Fig 4). In T1DM patients which carried TT-genotype of *PTPN22* gene, 93% had at least one DQ2 allele and 60% carried either a DQ2 or a DQ8 allele (Fig 4). The co-inheritance of HLA-DR alleles with *PTPN22* genotypes in T1DM patients has been presented in Fig 5. It showed that the variant TT genotype was detected together with three DR allele combinations, e.g. DR 3–5, 3–6 and 3–4 respectively. Although the heterozygous CT genotype occurred in association with other DR allele combinations but predominantly in those carrying either a DR3 or a DR4 allele (Fig 5).

In order to analyze gene-gene interaction, epistasis analysis was performed between *PTPN22* gene variant genotypes with HLA-DQ and HLA-DR genotypes using the case-only analytical approach since HLA-DQ and HLA—DR were genotyped in cases. Briefly, the case-only design is considered an efficient approach to test gene-gene interaction on multiplicative scale to assess if the disease risk among those who are jointly exposed is above and beyond the individual effects of each exposure [51]. Hence, the ‘susceptibility genes’ were dichotomized into two allelic variants (susceptible vs. non-susceptible). For the *PTPN22* gene, the heterozygote (CT) and variant homozygote (TT) genotypes were collapsed together and assumed to be the risk group and the wild-type genotype (CC) made the baseline/referent group. In the case of HLA-DQ, allelic combinations 1–2, 2–2, 2–3, 2–5, 2–8, and 8–8 were combined together to make the risk group. The HLA-DR allelic combinations 3–5, 3–3, 3–4, 3–7, and 4–4 were collapsed to make the risk group. Subsequently, we have assessed whether there is multiplicative interaction between *PTPN22* with HLA-DQ (Table 3) and *PTPN22* with HLA-DR (Table 4) on the risk of T1DM. Results showed that there was no gene-gene interaction between *PTPN22* and HLA-DQ on the risk of T1DM (case-only interaction OR = 1.05, 95% CI: 0.49–2.28; Table 3). In contrast, there was sufficient statistical evidence to indicate antagonistic interaction between *PTPN22* and HLA-DR on the risk of T1DM (case-only interaction OR = 0.22, 95% CI: 0.09–0.54; Table 4).

**Table 3:** Estimated case-only interaction odds ratio between *PTPN22* and HLA-DQ genotypes on the risk of T1DM.

*PTPN22* variants	HLA-DQ variants	Frequency of cases	Case-only OR_interaction_	95% CI	P-value
+	+	67	1.06	0.49–2.28	0.883
+	-	12			
-	+	116			
-	-	22			

+: Refers to the presence of risk alleles;−: refers to the absence of risk alleles; OR: odds ratio; CI: confidence interval.

**Table 4:** Estimated case-only interaction odds ratio between *PTPN22* and HLA-DR genotypes on the risk of T1DM.

*PTPN22* variants	HLA-DR variants	Frequency of cases	Case-only OR_interaction_	95% CI	P-value
+	+	19	0.22	0.09–0.54	< 0.001
+	-	34			
-	+	28			
-	-	11			

+: Refers to the presence of risk alleles;−: refer to the absence of risk alleles; OR: odds ratio; CI: confidence interval.

### Detection of three autoantibodies in T1DM patients, their correlation with *PTPN22* genotypes and age-of-onset of the disease

A total of 253 T1DM patients were screened for the presence of three auto-reactive antibodies ICA, GADA and INS. ICA-autoantibody was detected in 142/253 (56%) T1DM patients, GADA in 177/253 (83%) and INS was present in 177/253 (70%) patients. At least one autoantibody was detected in each T1DM patient. In the T1DM patients, 160 had CC genotype of the *PTPN22* gene, 76 had CT genotype and 17 T1DM patients were carrying the variant TT genotype. The data on autoantibody-positivity in T1DM patients carrying the three *PTPN22* genotypes has been presented in Table 5. The strongest correlation was detected in the case of GADA, followed by INS and ICA autoantibodies (Table 5).

**Table 5:** Correlation of autoantibody-positivity with genotypes of *PTPN22* gene functional variant (C1858C, rs2476601) in Kuwaiti TIDM patients. ICA, Islet cell autoantibody; GADA, glutamic acid decarboxylase autoantibody; INS, insulin autoantibody.

Autoantibody	Genotype/ Autoantibody Frequency CC (%)	Genotype/ Autoantibody Frequency CT (%)	Genotype/ Autoantibody Frequency TT (%)
**ICA**	87/160 (54)	44/76 (58)	11/17 (65)
**GADA**	130/160 (81)	62/76 (82)	17/17 (100)
**INS**	113/160 (71)	52/76 (68)	12/17 (71)

The correlation between presence of three autoantibodies, possession of *PTPN22* gene (rs2476601) variant genotypes and the age of onset of T1DM was also investigated and the data has been presented in Table 6. In the case of homozygous *PTPN22* variant (C1858T, rs2476601) genotype TT, the highest positive correlation was detected in the case of GADA autoantibody which was detected in 100% cases in all the three age-of-onset subgroups of T1DM patients. In T1DM patients with homozygous variant *PTPN22* genotype TT, the frequency of ICA and INS autoantibodies was lower in patients with age <4y compared to the other two age-of-onset subgroups (Table 6). In contrast, the frequency of ICA and INS autoantibodies was lower in patients who carried the heterozygous *PTPN22* genotype, CT in the case of age-of-onset 6-14y subgroup (Table 6). As mentioned earlier, one limitation of this analysis is the presence of low numbers especially in the case of T1DM patients who had the variant TT genotype which may which can potentially yield type II errors in making comparisons between the study groups.

**Table 6:** Correlation of autoantibody-positivity with genotypes of *PTPN22* gene functional variant (C1858T, rs2476601) in Kuwaiti TIDM patients stratified on the basis of age-of-onset of the disease. ICA, Islet cell autoantibody; GADA, glutamic acid decarboxylase autoantibody; INS, insulin autoantibody.

Age-of-onset group/ Autoantibody	Autoantibody/ Frequency Genotype		Autoantibody/ Frequency Genotype		Autoantibody/ Frequency Genotype	
	CC	(%)	CT	(%)	TT	(%)
**ICA Autoantibody**						
<4 years	23/34	(68)	7/10	(70)	2/4	(50)
4–6 years	23/40	(58)	11/15	(73.3)	6/8	(75)
>6 years	41/82	(50)	25/50	(50)	3/5	(60)
**GADA Autoantibody**						
<4 years	28/34	(82.4)	7/10	(70)	4/4	(100)
4–6 years	33/40	(83)	14/15	(93.3)	8/8	(100)
>6 years	69/82	(84)	40/50	(80)	5/5	(100)
**INS Autoantibody**						
<4 years	29/34	(85.3)	8/10	(80)	1/4	(25)
4–6 years	28/40	(70)	12/15	(80)	7/8	(88)
>6 years	56/82	(68.3)	31/50	(60)	4/5	(80)

### Correlation of three autoantibodies with HLA-DQ/DR genotypes in T1DM patients

**Fig 6:**
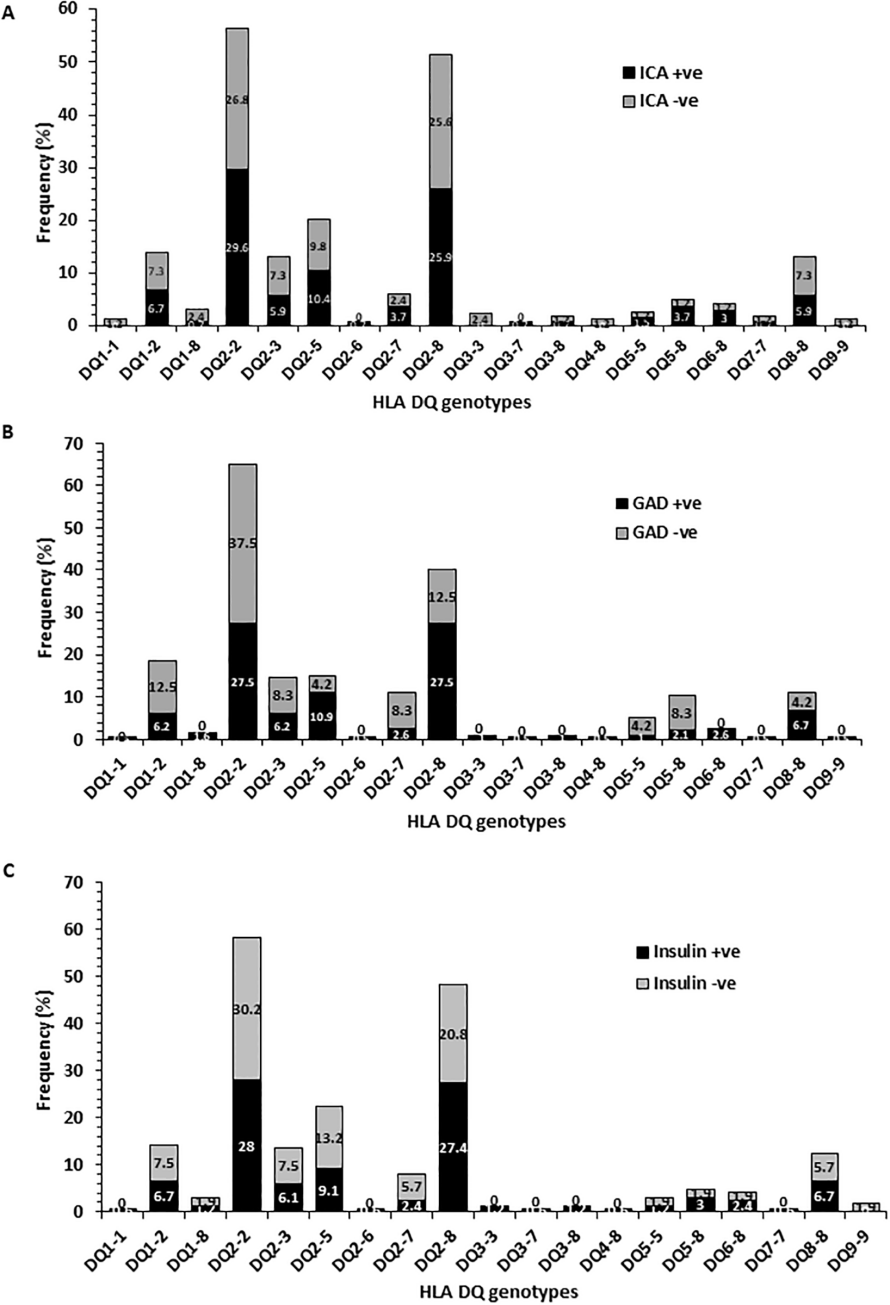
The correlation of HLA-DQ allele frequency in Kuwaiti T1DM patients with three autoantibodies. A) Islet-cell autoantibody; B) GADA autoantibody; C) INS autoantibody.

In T1DM patients the correlation between different HLA-DQ alleles and the presence of three autoantibodies was also investigated. These findings have been presented in Fig 6 (A-C). The ICA autoantibody was detected in nearly half of the Kuwaiti T1DM patients who harbored one of the ‘high risk’ DQ alleles (showing a marked association with T1DM, i.e. DQ 2–2, 2–8 and 8–8; Fig 6A). In contrast, the T1DM patients who carried a high risk DQ-8 allele, majority of patients were positive for GADA (Fig 6B). In T1DM patients with DQ 2–2, majority were negative for GADA (Fig 6B). The distribution of INS-autoantibody positive patients was also higher in T1DM patients having at least one DQ8 allele and autoantibody-positivity was slightly lower in patients with DQ2 alleles, Fig 6C.

**Fig 7:**
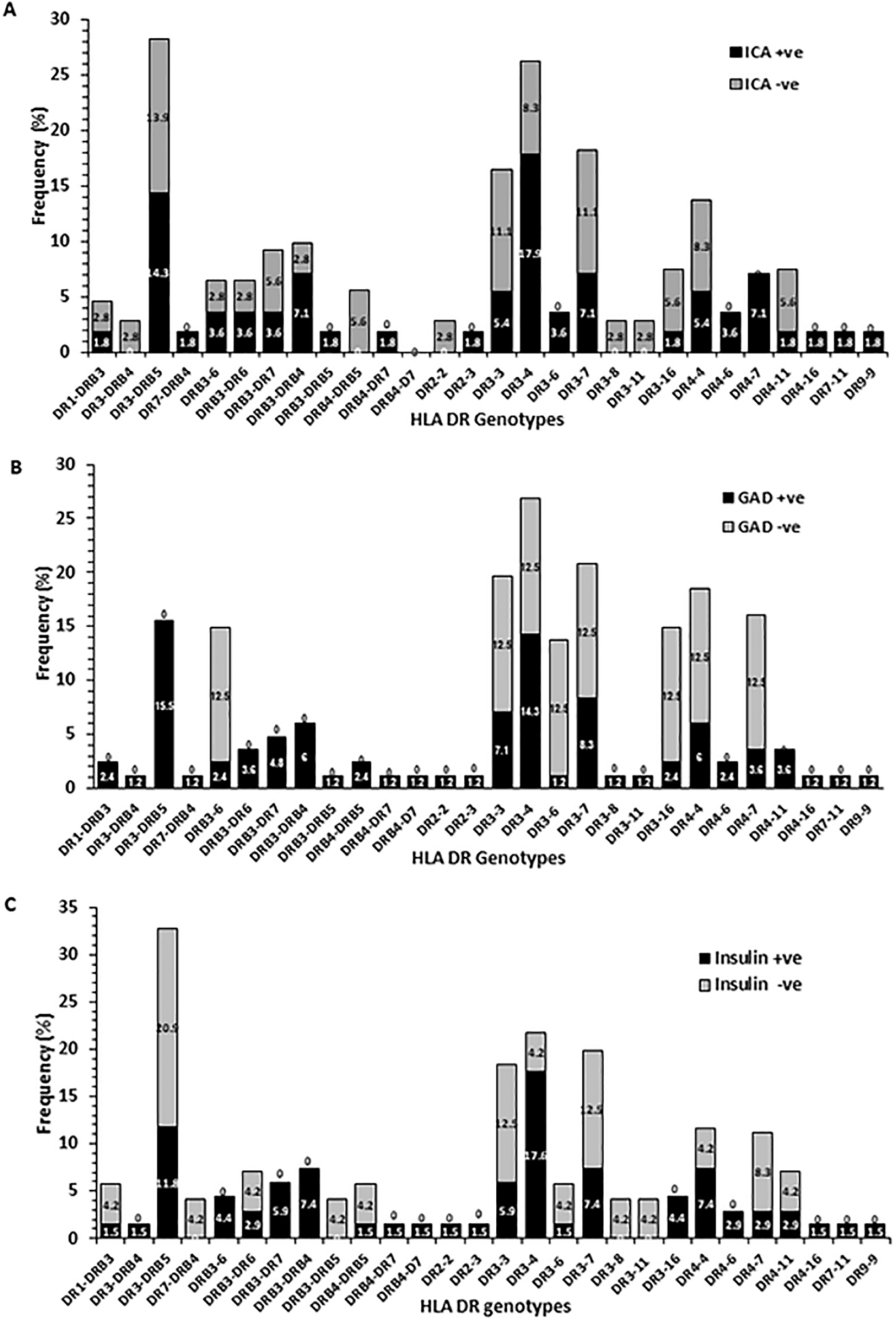
The correlation of HLA-DR allele frequency in Kuwaiti T1DM patients with three autoantibodies. A) Islet-cell autoantibody; B) GADA autoantibody; C) INS autoantibody.

In T1DM patients, the correlation between HLA-DR alleles and the presence of three autoantibodies was investigated and the data has been presented in Fig 7(A)–7(C). In the case of patients who carried the high risk HLA-DR alleles (DR-3-5, 3–4, 4–7 and DRB3-DRB4) majority of patients were positive for the ICA-autoantibody (Fig 7A). All the patients who harbored DR4-7 genotype were positive for ICA-autoantibody. GADA was detected in majority of the T1DM with genotypes DR3-DRB5; DR 3–4, and DRB3-DRB4 (Fig 7B). INS-autoantibodies were detected in majority of T1DM patients who carried the high risk DR alleles i.e. DRB3-DR7; DRB3-DRB4; DR3-4 and DR4-4 (Fig 7C). In the case of first two, all the patients were positive for INS autoantibody.

## Discussion

In this study, we report a significant association of the rs2476601 (C1858T) functional variant in the *PTPN22* gene with T1DM in Kuwaiti Arabs. Collectively, the variant T-allele was present in homozygous and heterozygous combinations in almost 37% of the Kuwaiti Arab T1DM patients. Although these results are generally consistent with several previous meta-analysis and case-control studies from European populations [18–24], the incidence of variant *PTPN22* genotype detected in Kuwaiti Arabs is amongst the highest. This is in contrast to a report from Greek T1DM patients, in which, no association was reported [52 45]. Also, some previous studies reported a lack of this variant in Asians (mainly from China and South Asia) [27–28]. A more recent report from China however, did find an association between the *PTPN22* gene and T1DM [53]. In Egyptian T1DM patients, the TT genotype was detected in only 2% patients [54]. Similarly, in T1DM patients from North India, although the frequency of T allele of the *PTPN22* gene was higher in T1DM patients (8%) compared to (2%) in the controls, none of the patients were homozygous for the TT genotype [55].

The most striking finding on the HLA-DQ allele was that DQ2 and DQ8 alleles were detected in 91% of the Kuwaiti T1DM patients thus highlighting them as the risk alleles. Amongst the HLA-DR alleles, DR3-DRB4, 3–3, 3–4, 3–7 and 4–4 had highest incidence in Kuwaiti T1DM patients. Therefore, in Kuwaiti T1DM patients, like some other populations/ethnic groups, the heterozygous combinations associated with a significantly high risk of disease onset are the DR3/DR4 and/or DQ2/8. Several previous studies report that in up to 50% cases, susceptibility is contributed to HLA DR and DQ [56–57]. Amongst the HLA-DQ and DR alleles, several high risk and protective alleles have been identified in previous literature [57–58] and our findings are generally consistent with these and with our earlier report from Kuwait [39]. It has been reported that HLA-DQ2/8 heterozygous individuals are at a considerably higher risk of developing type 1 diabetes possibly by expressing HLA-DQ8*trans* molecule on antigen-presenting cells as compared to the homozygous HLA-DQ2 or HLA-DQ8 individuals [59]. This has been attributed to qualitative and quantitative differences in recognition and presentation of the islet cell epitopes by the dendritic cells in individuals with DQ2, DQ8 or DQ2-8 genotypes during the autoimmune process [59].

Our data on the co-inheritance of *PTPN22* variant genotype (TT) and HLA-DQ alleles show that T1DM patients who had the homozygous variant *PTPN22* genotype (TT), always carried one or more of the high risk DQ alleles (DQ 2–2, 2–3, 2–5, 2–8 and 8–8, Fig 4). In Kuwaiti T1DM patients who carried the TT genotype, 93% had at least one DQ2 or a DQ8 allele. Similarly, the homozygosity for the variant *PTPN22* genotype was found to be associated with one or more of the ‘high risk’ HLA-DR alleles (Fig 5). This strongly suggest that the *PTPN22* gene variant (C1858T, rs2476601) along with specific HLA DQ/DR alleles (mentioned above) constitute strong risk factors in conferring susceptibility to T1DM in Kuwaiti Arabs. This is in contrast to some previous reports which showed that the strength of the association between T1DM and *PTPN22* gene variant (C1858T, rs2476601), decreased in patients with high risk genotypes e.g. HLA-DR3-DQ2/DR4-DQ8 [49, 60–61]. We carried out epistasis analysis to investigate gene-gene interaction between the *PTPN22* and HLA-DQ/DR genotypes with regard to their impact on susceptibility to T1DM. In the case-only analytical approach employed, our data (Tables 3 and 4) highlight these two candidate genes/loci as independent determinants for susceptibility to T1DM in Kuwait Arabs. This is not surprising because of the known biological role of these two loci in the auto-immune process; HLA being involved in presenting the auto-antigens while *PTPN22* gene product (LYP) serving as a key component of the intra-cellular signaling pathway [18].

Our data on association of three autoantibodies (ICA, GADA and INS) with *PTPN22* gene variant and the HLA-DQ/DR genotypes in Kuwaiti T1DM patients revealed an interesting and unique pattern. The incidence of positivity of all three autoantibodies followed a similar pattern in T1DM patients having three different *PTPN22* genotypes (CC, CT and TT). The most striking aspect of these correlations was that in T1DM patients who were homozygous for the variant *PTPN22* genotype (TT), ICA was positive in 65%, GADA in 100% and INS in 71% patients respectively. It may be added that the presence of low number of subjects in some of the groups compared may be considered as a limitation of our data. Previous studies have shown that GADA was associated with the HLA DR3-DQ2 haplotype and also with the variant *PTPN22* genotype [37, 62–63]. In another report, the association was primarily observed in older patients and the authors suggested that the prevalence of GADA was correlated with number of 1858C>T alleles in an additive, rather than dominant way [62]. In a study from Finland, the variant TT genotype of the *PTPN22* gene was associated with INS- autoantibodies [60]. Autoantibodies to pancreatic antigens have long been considered a characteristic feature of T1DM [57]. A recent study from India reported a low incidence of three autoantibodies i.e. GADA, ZnT8 and IA-2 in T1DM patients (only 47.06% patients had either one or a combination of these autoantibodies) with GADA being the most common autoantibody, detected in 38.23% cases [57]. It has been reported from the Western countries that nearly 85–90% of the newly diagnosed T1DM cases have GADA and/or IA-2 or ZnT8 or INS autoantibody [64–67]. A study from Saudi Arabia reported a high positivity of GADA (73.3%), followed by ICA (42%) and 27.3% patients had both these autoantibodies [68]. Chan et al. have found that in T1DM patients from China, GADA was detected in 31% cases [69]. In comparison to these, our data from Kuwaiti T1DM patients showed a higher incidence of GADA and INS autoantibodies; GADA was detected in 83%, INS in 70% and ICA in 56% of our patients. In Kuwaiti T1DM patients who carried the ‘high risk’ HLA-DQ alleles (DQ 2–2, 2–8 and 8–8), nearly half were positive for ICA and INS autoantibodies. GADA was positive in majority of Kuwaiti T1DM patients who had DQ 8–8 genotype but in contrast, majority of patients who had DQ 2–2 genotype were negative for GADA. It has been reported that children homozygous for HLA-DR3-DQ2 carried GADA as the first autoantibody [40–41] whereas children with HLA-DR4-DQ8 haplotype tended to have INS autoantibody in earlier phase of the disease [35, 42]. In the light of these, our results in T1DM patients with HLA-DQ 2–2 genotype appear to be unique and indicate that population specific effects can impact the risk-profile of markers when considered together. It is generally accepted that in children followed up from birth, because of increased HLA-DR-DQ genetic risk, such as in TEDDY [38], BABY DIAB [70–71], DIPP [72] and the Pre-POINT study [73], the environmental factors, non-HLA genes, or both increase the risk of seroconversion to either GADA-only in children with HLA-DR3-DQ2 or INS autoantibodies-only in children with HLA-DR4-DQ8 patients. Our results along with recent data from Next-generation sequencing studies [43] support the involvement of HLA genotypes as risk-modifiers and highlight the significance of simultaneous screening of multiple risk factors/markers in determining the genetic predisposition of T1DM in different populations/ethnic groups.

## Conclusions

The data reported in this study demonstrate that the variant T-allele of the *PTPN22* gene along with HLA-DQ2, DQ8, DR3 and DR4 alleles constitute significant determinants of genetic susceptibility to T1DM in Kuwaiti Arabs.

## Supporting information

S1 File PONE-D-17-42796-supporting info file. (SAV)
